# Exploring the Effects of Freeze-Dried Sourdoughs with *Lactiplantibacillus pentosus* 129 and *Limosilactobacillus fermentum* 139 on the Quality of Long-Fermentation Bread

**DOI:** 10.3390/microorganisms12061199

**Published:** 2024-06-14

**Authors:** Joanderson Gama Santos, Evandro Leite de Souza, Marcus Vinícius de Souza Couto, Tatiana Zanella Rodrigues, Ana Regina Simplício de Medeiros, Angela Maria Tribuzy de Magalhães Cordeiro, Marcos dos Santos Lima, Maria Elieidy Gomes de Oliveira, Maiara da Costa Lima, Noádia Priscilla Rodrigues de Araújo, Ingrid Conceição Dantas Gonçalves, Estefânia Fernandes Garcia

**Affiliations:** 1 Graduate Program in Agro-Food Technology, Federal University of Paraíba, Bananeiras 58220-000, PB, Brazil; joanderson.gama@gmail.com (J.G.S.); anareginasimplicio@gmail.com (A.R.S.d.M.); 2 Laboratory of Food Microbiology, Department of Nutrition, Federal University of Paraíba, João Pessoa 58051-900, PB, Brazil; maiaraclima@gmail.com; 3 Graduate Program in Nutrition Sciences, Federal University of Paraíba, João Pessoa 58051-900, PB, Brazil; marcussouza.inf@gmail.com (M.V.d.S.C.); tatiana.zanella@academico.ufpb.br (T.Z.R.); 4 Department of Food Technology, Federal University of Paraíba, João Pessoa 58051-900, PB, Brazil; atribuzycordeiro@gmail.com; 5 Department of Food Technology, Federal Institute of Sertão de Pernambuco, Petrolina 56302-100, PE, Brazil; marcos.santos@ifsertao-pe.edu.br; 6 Department of Nutrition, Federal University of Paraíba, João Pessoa 58051-900, PB, Brazil; mego@academico.ufpb.br; 7 Department of Gastronomy, Federal University of Paraíba, João Pessoa 58051-900, PB, Brazil; noadia@ctdr.ufpb.br (N.P.R.d.A.); ingridcdantas@ctdr.ufpb.br (I.C.D.G.); estefaniafgarcia@yahoo.com.br (E.F.G.)

**Keywords:** *Lactobacillus* spp., bioactive compounds, bakery, freeze-drying, backslopping, fermentation

## Abstract

Sourdough production is a complex fermentation process. Natural sourdough fermentation without standardization causes great variability in microbial communities and derived products. Starter cultures have emerged as alternatives to natural fermentation processes, which could improve bakery quality and produce bioactive compounds. This study aimed to evaluate the impacts of freeze-drying on the production and viability of sourdoughs with *Lactiplantibacillus pentosus* 129 (Lp) and *Limosilactobacillus fermentum* 139 (Lf), as well as their effects on the quality of long-fermentation bread. These strains were selected based on their better performance considering acidification and exopolysaccharide production capacity. Sourdough with Lp and Lf were propagated until the 10th day, when physicochemical and microbiological parameters were determined. The produced sourdoughs were freeze-dried, and bread samples were produced. The freeze-drying process resulted in high survival rates and few impacts on the metabolic activity of Lp and Lf until 60 days of storage. Incorporating Lp and Lf improved the microbiological and physicochemical properties of sourdough and long-fermentation breads. Tested freeze-dried sourdoughs led to reduced bread aging (higher specific volume and decreased starch retrogradation) and increased digestibility. The results show the potential of the freeze-dried sourdoughs produced with Lp and Lf as innovative strategies for standardizing production protocols for the bakery industry, especially for producing long-term fermentation bread.

## 1. Introduction

Sourdough production is a complex fermentation process involving different microorganisms, such as lactic acid bacteria (LAB) and yeasts, which play an important role in bread production. This microbial environment in a mixture of flour and water generates carbon dioxide to leaven the dough and produces several compounds contributing to flavor, acidification, proteolysis, and inhibition of spoilage fungi [1,2].

The diversity of LAB in sourdough is an important factor in distinguishing it from bread fermented solely by *Saccharomyces cerevisiae*. LAB produces organic acids [2,3] and exopolysaccharides (EPS) with bioactive properties, in addition to acting as emulsifying and stabilizing agents to influence the technological and sensory characteristics of bread [2,4,5]. LAB, such as *Lactiplantibacillus pentosus* and *Limosilactobacillus fermentum,* are commonly associated with EPS production [3,6,7].

Dominant microbial species during sourdough and bread fermentation lead to variations in the physicochemical characteristics of the obtained product. Natural sourdough fermentation without strict control of temperature, pH, and incubation time results in pronounced variability in the microbial community forming the sourdough ecosystem, which impacts the quality of the obtained products [7,8]. The high costs associated with standard sourdough maintenance and the absence of standardized production protocols impose significant challenges for the bakery industry [2,9,10].

The sourdough market was estimated at USD 2.5 billion in 2018, with a predicted annual growth rate of 5.7%, reaching USD 3.5 billion by 2025. Europe accounted for 30.4% of the global sourdough market in 2018. The bakery industry is expected to grow mostly due to increasing demand for preservative-free products and the high availability of raw materials [10]. However, sourdough with these characteristics remains a challenge, particularly with liquid sourdough that requires successive propagations [10,11].

Starter cultures have emerged as alternatives to sourdough processes [11,12], and drying techniques, such as freeze-drying and spray-drying, have been used to preserve these cultures [13,14]. A previous study showed that incorporating freeze-dried-specific strains of *Lactipantibacillus plantarum* and *Lactococcus lactis* improved the dough viscoelasticity, aroma, and bread acidity [15]. Several studies have evaluated the use of dried sourdough inoculated with different LAB species [16,17,18,19]. These drying technologies could stabilize dried sourdough and preserve starter microorganisms, assisting in maintaining dough vitality and sensory characteristics in bread production [18]. However, in all cases, the viability of the yeasts forming the sourdough microbiota is sharply reduced, and commercial baker’s yeast is used in the sourdough or directly incorporated in the dough to promote increased volume [18].

The application of cryoprotectants during freeze-drying helps to maintain the viability of beneficial dough microorganisms and keeps the production of desired metabolites [20]. Previous investigations have evaluated using skimmed milk, whey protein, disaccharides, and fructooligosaccharides (FOS) to decrease the damage of starter culture cells and produce standard powdered sourdoughs [2,10,16,21]. Especially, FOS have been shown to be effective in protecting bacterial cell membranes [22] and acting as a stabilizing agent during freeze-drying of bacterial cultures and storage [20]. Powdered sourdough could present a longer shelf life, consistent product quality, ease of formulation, and reduced transportation costs [19]. However, there is still scarcity in the market of dried sourdough capable of enhancing flavor and promoting volume increase in bread without the need to add baker’s yeast.

This study evaluated the impacts of *L. pentosus* 129 and *L. fermentum* 139 strains individually as starter cultures in the production of freeze-dried sourdough and their effects on physicochemical parameters, digestibility, and aging characteristics of long-fermentation bread. In addition, the viable cell counts and the physiological status of *L. pentosus* 129 and *L. fermentum* 139, as well as the physicochemical characteristics of produced sourdough, were evaluated.

## 2. Materials and Methods

### 2.1. Microorganisms, Growth Conditions, and Inoculum Preparation

Five strains of *Lactobacillus* spp., which were isolated and previously identified [23,24], were initially screened in this study regarding acidification activity and exopolysaccharide production as important characteristics for sourdough development and bread production [2,4,5] (Table S1). The strains were stored at −18 °C in de Man, Rogosa, and Sharpe (MRS) broth (Sigma Aldrich, Darmstadt, Germany) supplemented with 80% glycerol (Sigma Aldrich). For bacterial inoculum preparation, each strain was cultivated anaerobically (Anaerogen System Anaerogen, Oxoid, Hampshire, UK) in MRS broth until reaching the stationary phase (24–36 h, 30 ± 1 °C), collected by centrifugation (6000 rpm, 5 min, 4 °C), washed twice in phosphate-buffered saline solution (PBS, for 1 L: 8 g NaCl, 0.2 g KCl, 1.4 g Na_2_HPO_4_, 0.2 g KH_2_HPO_4_, pH 7.4), and the bacterial inoculum was standardized to an optical density at 625 nm of 1.0, which provided viable cell counts between 10–11 log colony-forming units per milliliter (log CFU/mL) when plated on MRS agar and incubated aerobically for 48 h at 30 ± 1 °C (Sigma Aldrich).

### 2.2. Characterization and Selection of Lactobacillus spp. Strains

#### 2.2.1. Acidification Activity

The acidification activity of the *Lactobacillus* spp. strains was examined in vitro using sterile flour extract (SFE) broth [25]. The fresh bacterial inoculum of each strain was added (1%, *v*/*v*) into 20 mL of SFE broth and incubated for 72 h at 30 ± 1 °C. The broth pH was measured at 2, 4, 6, 8, 24, 48, and 72 h of incubation [26].

#### 2.2.2. Exopolysaccharide (EPS) Production and Extraction

LAB strains were grown in MRS broth supplemented with glucose (2%, *w*/*v*) for 3 days at 37 ± 1 °C under anaerobic conditions (Anaerogen System Anaerogen). The cells were centrifuged (6000 rpm, 20 min, 20 °C), mixed in a 1:2 ratio with 95% (*v*/*v*) cold ethanol (Fmaia, Belo Horizonte, MG, Brazil), and stored at 4 °C for 24 h to induce EPS precipitation. The precipitated EPS was recovered with centrifugation (4000 rpm, 15 min, 4 °C), washed with sterile distilled water, and dried at 55 °C until reaching a constant weight. The dry weight concentration (mg/L) was measured to determine the content of EPS produced [6].

### 2.3. Preparation of Sourdough

Based on the better combination of acidification and EPS production capacity, a Principal Component Analysis (PCA) determined the most promising *Lactobacillus* spp. strains for sourdough production, and the strains *L. pentosus* 129 (Lp) and *L. fermentum* (Lf) were selected (Figure S1). The inoculum of each tested strain was added to the respective sourdough in the proportions: Wheat flour: 168.75 g, water: 101.25 mL, and inoculum: 10 mL [27]. The samples included: (i) sourdough with Lp (SLp); (ii) sourdough with Lf (SFf); and (iii) sourdough prepared under the same conditions but without adding Lp or Lf (Sc). The sourdough was propagated and incubated in a Bio-Oxygen Demand (BOD) incubator (28 ± 1 °C, 24 h) until maturation (10 days), with viable cell count enumeration every 24 h.

During the 10 days of propagation, the first back-slopping stage was initiated by taking a portion at the same proportions (1:1:1) of the 24 h-incubated sourdough and mixing it with potable water and flour (Dona Benta, Moinho J. Macêdo, Salvador, BA, Brazil). This back-slopping process was uniformly performed for all sourdough samples (SLp, SFf, and Sc), followed by incubation at 30 ± 1 °C for 24 h and repeated every day for 10 days.

### 2.4. Sourdough Freeze-Drying

On the 10th day of propagation, the sourdough samples (SLp, SLf, and Sc) were supplemented with FOS (10%, *w*/*w*) as a cryoprotectant. The samples were frozen (−18 °C, 24 h) and subsequently freeze-dried (temperature of −55 ± 2 °C, vacuum pressure of <138 μHg, freeze-drying rate 1 mm/h, approximately 40 h) using a bench-top freeze-dryer (Liotop, Model L-101, São Carlos, SP, Brazil). The powder of each sourdough sample was packed in polyethylene bags, vacuum-sealed, and stored at room temperature (28 ± 1 °C) for 60 days. Freeze-dried sourdough samples (FSLp, FSLf, and FSc) were measured at zero, 15, 30, 45, and 60 days of storage.

### 2.5. Determination of the Physiological Status of the Freeze-Dried Sourdough Microbiota

The physiological status of the microbiota in sourdough samples was analyzed immediately after freeze-drying using multiparametric flow cytometry (MFC). Initially, the samples were centrifuged (6500 rpm, 10 min, 4 °C), washed twice, and resuspended in sterile PBS. The MFC analyses were performed using a flow cytometer with an argon ion laser emitting at 488 nm (BD Accuri C6, Becton Dickinson, Franklin Lakes, NJ, USA). The FL1 channel [533 nm ± 30 nm, carboxyfluorescein diacetate (cFDA) staining] and FL3 channel [>670 nm, propidium iodide (PI) staining] collected the green and red fluorescence, respectively. Threshold levels for data acquisition were set on the forward scatter (FSC) (12,000) and side scatter (SSC) (30,000) to eliminate particles much smaller than intact cells and delimit the bacterial cells, respectively.

The concentration of cells in fresh and freeze-dried sourdough was measured using a low flow rate setting, and a total of 10,000 events were analyzed. Data analysis was performed using BD Accuri C6 Software (Becton Dickinson and Company, Franklin Lakes, NJ, USA). For the dual staining with PI and cFDA, the cell subpopulations characterized as PI-cFDA+ were considered non-permeabilized cells with enzymatic activity, while the cell subpopulations characterized as PI+cFDA- were considered permeabilized cells without enzymatic activity [28,29,30].

### 2.6. Long-Fermentation Bread Production

Portions of each freeze-dried sourdough sample (FSLp, FSLf, and FSc) were used to produce bread loaves, as described in Table S2. All ingredients were weighed according to the proportions shown in Table S3 [16]. The dry components, including wheat flour (type 1, Dona Benta), salt (Veneza, Reprasal, Mossoró, RN, Brazil), and sugar (Petribu, Lagoa de Itaenga, PE, Brazil), were combined with water and sourdough. None of the formulations were supplemented with baker’s yeast.

The dough was mixed in a bread machine (Britânia Multi Pane, Joinville, SC, Brazil) for 5 min. Butter (Camponesa, Embaré, Lagoa da Prata, MG, Brasil) was added to the dough after 3 min once the gluten was partially developed. After kneading, the dough underwent the first fermentation for 20 min with folds every 10 min to develop a soft, elastic, and extensible dough. Following the initial fermentation, the dough was weighed, divided into 100 g portions, rounded, and shaped into loaves before being immediately placed on baking sheets dusted with wheat flour for the second fermentation. In the post-second fermentation, the loaves were transferred to molds and baked in a bakery batch oven at 200 °C for 30 min. After removal from the oven, the loaves were cooled to room temperature (28 ± 1 °C), packed in polyethylene bags, labeled, and stored (28 ± 1 °C). The microbiological and physicochemical parameters of bread samples were assessed at 0, 1, 3, and 7 days of storage. Bread samples were named BFSLp (bread with FSLp), BFSLf (bread with FSFf), and BFSc (bread with FSc).

### 2.7. Physicochemical and Microbiological Parameters of Sourdough and Long-Fermentation Bread during Storage

#### 2.7.1. Enumeration of LAB and Yeast Viable Cells

The LAB and yeast viable cell counts in sourdough (SLp, SLf, and Sc) were enumerated at day zero and daily throughout the propagation period (from day 1 to 10); in freeze-dried sourdough (FSLp, FSLf, and FSc), they were enumerated at zero (immediately after freeze-drying), 15, 30, 45, and 60 days of storage [18]. To LAB enumeration, 25 g of the sourdough sample was diluted in 225 mL of 0.1% (*w*/*v*) peptone water solution and serially diluted (1:9, *v*/*v*) in the same diluent (10^−2^–10^−6^). Aliquots of 20 µL from each serial dilution were inoculated (using the micro-drop method) onto plates containing MRS agar and incubated aerobically at 37 ± 1 °C for 48 h [31,32]. To determine the yeast viable cell count, aliquots of 100 µL from each serial dilution were inoculated onto plates containing Potato Dextrose Agar (PDA, Sigma Aldrich) and incubated at 28 ± 1 °C for 72–96 h [16]. After the incubation period, the viable cells were enumerated, and the results were expressed as log CFU/g.

#### 2.7.2. Determination of Total Titratable Acidity, pH, Water Activity, and Moisture

To determine the total titratable acidity (TTA), 10 g of the examined bread or sourdough was homogenized into 90 mL of distilled water. Two drops of phenolphthalein solution were added to the suspension and titrated with sodium hydroxide solution (NaOH, 0.1N). TA was expressed as mL of 0.1N NaOH consumed per 10 g of the sample [33]. The same suspension was used to measure the pH using a potentiometer (model PHS-3E, Even, São Paulo, SP, Brazil) previously calibrated and operated according to the manufacturer’s instructions [33]. Water activity (a_w_) was measured using an a_w_ meter (Aqualab, Decagon Devices, Pullman, WA, USA) at a constant temperature (24 ± 1 °C). Moisture content was determined by drying 2 g of each bread sample in a convection oven (ACB Labor, São Paulo, SP, Brazil) at 105 °C until reaching constant weight [33].

#### 2.7.3. EPS Quantification

The quantification of EPS in freeze-dried sourdough was performed immediately after freeze-drying [34]. Twenty grams of each sample was diluted with distilled water (1:2, *v*/*v*), the suspension was centrifuged (6600 rpm, 15 min, 4 °C), two volumes of cold ethanol were added to the supernatant, and the sample was left to rest at 4 °C for 24 h. The precipitate was collected by centrifugation (4500 rpm, 20 min, 5 °C), the EPS was dissolved (1:2, *v*/*v*) in distilled water, two volumes of ethanol were added to the suspension, and centrifuged (4500 rpm, 20 min, 5 °C). Finally, the precipitates were dried in an oven, and the produced EPS was calculated using a gravimetric method (mg/g).

#### 2.7.4. Determination of Sugars and Organic Acids

The contents of sugars and organic acids in freeze-dried sourdough samples were quantified immediately after freeze-drying. Initially, 2 g of each freeze-dried sourdough was homogenized in 10 mL of ultrapure water (MilliQ^®^ system, EMD Millipore, Burlington, MA, USA) for 10 min using a mini Turrax. The samples were centrifuged (6000 rpm, 15 min, 4 °C) and filtrated through a 0.45 μm Millex-HA filter (Millipore Co., Bedford, MA, USA). The analyses were carried out using the high-performance liquid chromatography (HPLC) technique with an Agilent chromatography system (model 1260 Infinity LC, Agilent Technologies, Santa Clara, CA, USA) coupled with a refraction index detector (RID) and a diode array detector (DAD). An Agilent Hi-Plex H column (7.7 × 300 mm, 8 μm) and 4 M H_2_SO_4_ in ultrapure water were the mobile phase (flow rate 0.5 mL/min). The data were processed using the OpenLAB CDS ChemStation EditionTM software (Agilent Technologies). Standard curves and quantitative analysis were performed for each compound.

#### 2.7.5. Determination of Bread-Specific Volume and Color

The specific volume of the bread loaves was determined using the displacement method of millet seeds (*Panicum miliaceum* L.), and the results were expressed as g/cm^3^ [17]. The color of the bread crumb was assessed at three points on central slices measuring 3 × 3 cm. The measurements were taken using a CR-300 colorimeter (Minolta, Osaka, Japan) and color parameters, namely L*, ranging from zero (black) to 100 (white); a*, indicating a range from red (positive) to green (negative); and b*, indicating a spectrum from yellow (positive) to blue (negative), were determined. These parameters were analyzed according to the CIE Lab (Lab*) method [16].

#### 2.7.6. Texture Analysis

Texture parameters were determined with five replicates using the double compression test with a CT3 Texture Analyzer texturometer (Brookfield, Middleborough, MA, USA) equipped with a 25 mm diameter cylindrical compression probe. The bread loaf samples were cut into slices of 1.5 cm thickness, 3 cm height, and 3 cm width and subjected to texture analysis under the following conditions: Pre-test speed 5 mm/s, test speed 1.0 mm/s, and post-test speed 5 mm/s, 30% compression and time between the two compressions of 5 s. The measured parameters were hardness, chewiness, and elasticity [35].

#### 2.7.7. Differential Scanning Calorimetry Analysis

Differential scanning calorimetry (DSC) was carried out simultaneously using previously calibrated NETZSCH equipment (model STA 449F3). Ten mg of each sourdough bread sample was placed in an aluminum crucible under a nitrogen atmosphere (50 mL/min) and heated at 10 °C/min from room temperature to 1000 °C. The analyzed parameters were initial temperature (T0), peak temperature (Tp), final temperature (Tf), and enthalpy (ΔH) of retrogradation starch [36], and differential scanning calorimetry, which were measured at days 0 and 6 of storage.

#### 2.7.8. Determination of Bread In Vitro Digestibility

One gram of each bread sample was incubated with 1.5 mg of pepsin in 15 mL of 0.1 M HCl at 37 ± 1 °C for 3 h. After neutralization with 2 M NaOH and adding 4 mg of pancreatin in 7.5 mL of PBS (pH 8.0), 1 mL of toluene was added to prevent microbial growth, and the solution was incubated for 24 h at 37 ± 1 °C. After 24 h, the enzyme was inactivated by adding 10 mL of trichloroacetic acid (20%, *w*/*v*), and the undigested protein was precipitated. The volume was made up to 100 mL with distilled water, and the mixture was centrifuged (5000 rpm, 20 min, 4 °C). Protein concentration in the supernatant was determined using the value detected in the sample before digestion (blank) and an equation provided to calculate the in vitro protein digestibility [37,38].

### 2.8. Statistical Analysis

All assays were performed in triplicate on three independent experiments, and the results were expressed as average ± standard deviation. The Kolmogorov–Smirnov test checked the data’s normal distribution. Significant differences were determined (*p* < 0.05) with ANOVA followed by Tukey’s test. Pearson’s correlation tests were performed to explore relationships among dependent variables, while Principal Component Analysis (PCA) was performed to identify the most promising strains based on acidification capacity and EPS production. A *p*-value of <0.05 was considered statistically significant. The XLSTAT program version 2019.2.2.5964 (Lumivero, Denver, CO, USA) was used for the statistical analysis.

## 3. Results

The acidification capacity in sterile flour extract (SFE) for the examined *Lactobacillus* spp. strains showed pH values ranging from 5.58 ± 0.12 to 6.67 ± 0.04 at time zero, which decreased to a range of 3.42 ± 0.11 to 3.58 ± 0.03 at 72 h (Table 1). At 6 h, pH values dropped to below 5 for Lp 129, Lf 139, and Lf 141, and dropped to below 4 at 24 h, except for Lf 56 and control. At 72 h, all strains showed pH values below 3.60, while Lp 129, Lf 139, and Lf 141 showed the lowest pH values.

The tested *Lactobacillus* spp. strains showed EPS production capacity varying from 0.13 ± 0.01 to 0.33 ± 0.03 mg/L, with Lf 56 and Lf 141 showing the highest and lowest EPS production capacity, respectively (data now shown). LAB viable cell counts in the sourdough samples varied from <2 to 9.19 ± 0.02 log CFU/g during the measured back-slopping time (Table 2). Changes in LAB and yeast viable cell counts were observed between the third and eighth day of backslopping. However, all samples had increased LAB viable cell counts on the ninth and tenth days of propagation (backslopping).

Yeast viable cell counts varied from 1.00 ± 0.07 to 7.20 ± 0.16 log CFU/g for the samples over the measured backslopping (propagation) time (Table 2). The highest yeast viable cell counts were detected on the 10th day of backslopping for all sourdough samples. After the fourth day, all sourdough samples had reduced yeast viable cell counts (<4 log CFU/mL). Yeast viable cell counts stabilized by the ninth day of backslopping with no difference between the evaluated sourdough samples (*p* ≥ 0.05). Notably, only characteristic yeast colonies were observed throughout the measured backslopping period for all sourdough samples, indicating that filamentous fungi were inhibited, likely due to the decreasing pH in sourdough samples over time.

Table 3 shows the LAB and yeast viable cell counts (log CFU/g) in sourdough samples before and after freeze-drying, as well as during 60 days of storage. LAB viable cell counts ranged from 9.08 ± 0.60 to 8.72 ± 0.04 log CFU/g before freeze-drying, while the viable cell counts ranged from 8.64 ± 0.04 and 6.70 ± 0.02 log CFU/g after freeze-drying and during the 60 days of storage. The yeast viable cell counts in sourdough samples before freeze-drying ranged from 7.35 ± 0.02 to 7.29 ± 0.02 log CFU/g and ranged from 7.20 ± 0.13 to 5.00 ± 0.03 log CFU/g after freeze-drying and during 60 days of storage. After freeze-drying, examined sourdough samples had a reduction in LAB and yeast viable cell counts of <0.6 log CFU/g, but no significant difference (*p* ≥ 0.05) was observed in LAB or yeast viable cell counts before and after freeze-drying. A reduction of approximately 1 log CFU/g in LAB viable cell counts was observed only at day 60 of storage, reaching viable cell counts between 6–7 log CFU/g. Similarly, the yeast viable cell counts were reduced by 1–2 log CFU/g in freeze-dried sourdough samples at 60 days of storage.

**Figure 1:**
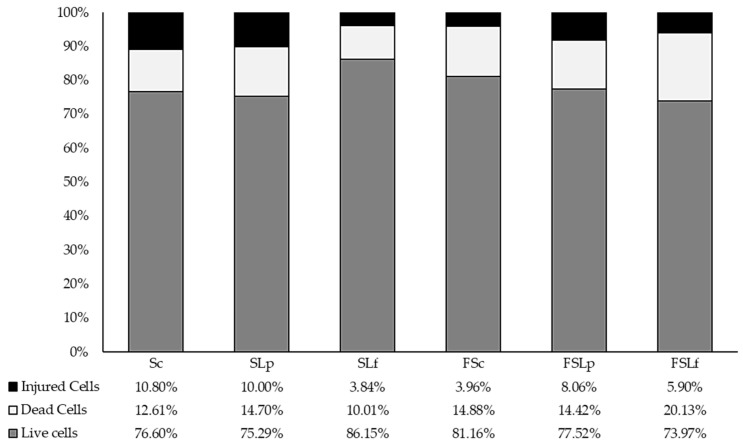
Sizes of microbial cell subpopulations with different physiological status in fresh sourdough before and after the freeze-drying. Sc: sourdough control; SLp: sourdough inoculated with *L. pentosus* 129; SLf: sourdough inoculated with *L. fermentum* 139; FSc: freeze-dried sourdough control; FSLp: freeze-dried sourdough inoculated with *L. pentosus* 129; FSLf: freeze-dried inoculated with *L. fermentum* 139.

Figure 1 shows the size of microbial cell subpopulations with different physiological statuses (live, injured, and dead) in sourdough before and after freeze-drying. The populations of live microbial cells were greater than 70% in sourdough samples before and after freeze-drying. Among fresh sourdough samples (before freeze-drying), the greater populations of live microbial cells were detected in SLf (86.15%), Sc (76.60%), and SLp (75.29%), respectively. Immediately after freeze-drying, the greater populations of live microbial cells were detected in FSc (81.16%), FSLp (77.52%), and FSLf (73.97%), respectively. The highest reduction in the live microbial cell population after freeze-drying was detected in FSLf (12.18%).

Table 4 shows the results for the physicochemical parameters of freeze-dried sourdough during storage. The pH values increased, while the TTA values reduced, especially for the control sample. The moisture (3.66 ± 0.04 to 7.96 ± 0.06) and a_w_ (0.18 ± 0.00 to 0.48 ± 0.02) of freeze-dried sourdough increased during storage, but these variations seemed not to vary with the type of the inoculated strain.

The EPS contents in the freeze-dried sourdough samples ranged from 0.27 ± 0.1 mg/L (FSc) to 0.33 ± 0.13 mg/L (FSLf), with no significant differences between sourdough samples (*p* ≥ 0.05). The contents of sugar and organic acids were determined immediately after freeze-drying sourdough samples (Table 5). Fructose was the sugar with the higher contents in sourdough samples, ranging from 3.04 ± 0.09 mg/L (FSLp) to 5.30 ± 0.1 mg/L mg/L (FSLf). Glucose contents ranged from 1.20 ± 0.19 mg/L (FSLf) to 3.90 ± 0.16 mg/L (FSc) in sourdough samples.

The tested strains did not impact the contents of glucose and fructose in freeze-dried sourdough (*p* ≥ 0.05). The content of maltose in FSLf was 0.61 ± 0.11 mg/L, while it was below the detection limit in FSc and FSLp. Acid organic quantification showed that FSLf had a different behavior from the other examined sourdough samples, with a higher content of lactic acid (0.70 ± 0.13 mg/L), probably associated with a more intense metabolic activity of Lf. The lowest contents of succinic acid were detected in FSLf (0.03 ± 0.09 mg/L), followed by FSLp (0.30 ± 0.16), with the highest amount detected in FSc (1.43 ± 0.25).

Table 6 shows the values of the physicochemical parameters (TTA, pH, moisture, specific volume, and color) and determination of bread staling (texture and differential scanning calorimetry, i.e., heat of retrograded starch fusion, ∆H) of long-fermentation bread made with freeze-dried sourdough inoculated or not with FSLp or FSLf during 6 days of storage.

The pH and TTA values in the examined breads ranged from 4.43 ± 0.06 to 4.38 ± 0.02 and 3.43 ± 0.15 to 2.71 ± 0.15 mL NaOH/10 g during storage, respectively. Overall, the pH values did not change during storage (*p* ≥ 0.05), but breads with BFSLp and BFSLf were more acid at time zero (*p* < 0.05). The moisture values were reduced regardless of the tested bread sample (*p* < 0.05); however, especially on day 6 of storage, the bread produced with FSLp had the highest moisture (*p* < 0.05). The specific volume ranged from 3.22 ± 0.16 to 2.65 ± 0.14 cm^3^/g among the examined bread samples. A higher specific volume was detected for bread produced with FSLf, ranging from 3.12 ± 0.04 to 2.99 ± 0.08 cm^3^/g.

The results of color parameters in the crumbs of bread produced with the freeze-dried sourdoughs ranged from 76.63 ± 0.84 to 79.82 ± 0.78 for L* parameter, from 0.44 ± 0.11 to 0.68 ± 0.01 for a* parameter, and from 14.40 ± 0.57 to 16.38 ± 0.87 for b* parameter. On day 6 of storage, the samples had a decrease in L* values, except for bread produced with FSLf. The hardness (H) increased in all the bread samples during storage; however, bread produced with FSLf had the lowest hardness at each storage time (zero, 3, or 6 days). BFSLf had the lowest chewiness (C) values (*p* < 0.05) at days 0 and 6 of storage compared to other bread samples. Bread produced with FSLf had reduced hardness, as well as less impact on the elasticity of bread loaves on day 6 of storage, contributing to reduced aging.

Differential scanning calorimetry (DSC) characterized retrograded starch after baking bread made with freeze-dried sourdoughs. The evaluated parameters were the melting heat of retrograded starch (H), initial transition temperature (T0), peak transition temperature (Tp), and final transition temperature (Tf) (Supplementary material data, Table S5). The initial transition temperature in the bread samples ranged from 26.4 to 28.3 °C; the values of the peak transition temperature ranged from 33.9 to 37.1 °C; the values of the final transition temperature ranged from 49.8 to 55.5 °C (Supplementary material data, Table S5). The lowest enthalpy value at day 6 of storage was detected in bread produced with FSLf (61.10 J/g), followed by bread produced with FSLp (73.2 J/g), and control (86.2 J/g). Results of the texture evaluation and DSC assays indicate that using FSLf in bread production reduces the aging action during storage.

All bread loaves produced with freeze-dried sourdoughs had a reduced protein content after in vitro digestion. Tested bread loave samples had in vitro digestibility > 90%; however, bread produced with FSLp and FSLf had a higher in vitro digestibility (98.6 and 98.1%, respectively) than the control (91.1%) (*p* ≥ 0.05).

## 4. Discussion

The fermentation of sourdough results from a complex fermentation process involving different microorganisms. The microbial environment can improve bread production, contributing to flavor, acidification, proteolysis, and inhibition of spoilage fungi, resulting in distinct physicochemical, microbiological, and sensory characteristics in the produced fermented bread [9,10]. In the present study, the effects of *L. pentosus* 129 (Lp) and *L. fermentum* 139 (Lf) individually as starter cultures in the production of freeze-dried sourdough, as well as their effects on physicochemical parameters, digestibility, and aging characteristics of long-fermentation bread, were described.

Microorganisms used in natural fermentation must possess certain characteristics to adapt to both the fermentation conditions and the matrix where are introduced [10,39]. The capacity to produce organic acids and tolerate pH fluctuations throughout the fermentation process are key attributes for strain selection. In addition, acidification and production of secondary metabolites by LAB, such as EPS, contribute to aroma, flavor, texture, and dough development in sourdough bread production [40]. In this study, different LAB strains were initially tested (Lf 56, Lpc 106, Lp 129, Lf 139, and Lf 141), aiming to select those with the better acidification and EPS production capacity when the strains Lp and Lf were chosen for sourdough production.

The high viable cell counts of Lp and Lf in sourdough reinforced their fermentation capacity; in parallel, the high yeast viable cell counts also detected indicated the incorporation of wild microbiota in the produced sourdoughs. Backslopping results could be mostly linked to the behavior of the best-adapted strains in the sourdough environment [41], indicating that the selected strains (Lp and Lf) could successfully adapt to this complex environment.

The freeze-drying process caused few impacts on the viable cell counts of sourdough during 60 days of storage. Previous investigations reported reductions in LAB and yeast viable cell counts after freeze-drying, probably due to cell thermal stress caused by freezing processes [16]. However, the FOS incorporation could protect microorganisms against damage produced by freeze-drying [42,43]. Multiparametric flow cytometry was used to investigate the physiological status of the freeze-dried sourdough microbial populations, confirming the results of viable cell counts. The results showed populations of live microbial cells greater than 70% in sourdough samples before and after freeze-drying. An early study reported the association between EPS synthesis and greater cellular resistance to stress caused by freeze-drying *Lactobacillus acidophilus* NCFM [44]. Studies developing freeze-dried sourdough inoculated with LAB having yeasts from wild microbiota and capable of maintaining high populations of viable microorganisms to promote volume increase in bread without baker’s yeast are still scarce, especially considering the production, reactivation, and fermentation protocols adopted in this study.

The measurements of physicochemical parameters of freeze-dried sourdough during 60 days of storage showed some variations in pH and TTA values over time. The increase in moisture and a_w_ over time is expected for dried products; however, powders with moisture between 2 and 8% and a_w_ below 0.60 are considered safe for the non-development of spoilage microorganisms [45].

The assessment of the sugar and organic acid profile of freeze-dried sourdough indicated a more intense metabolic activity of Lf (FSLf) linked to a decrease in the contents of sugars (fructose, glucose, and maltose) and an increase in the contents of organic acids (especially lactic acid) during storage. In addition, results indicated a metabolization of maltose in FSLp. Some heterofermentative LAB synthesizes the enzyme maltose phosphorylase when well adapted to sourdough. This enzyme allows the breakdown of maltose, flour’s most abundant energy source, into glucose and 1-P glucose without using ATP. The excretion of non-phosphorylated glucose induces glucose repression in other microorganisms in the sourdough ecosystem, preventing competition for maltose [45,46]. Using alternative electron acceptors in the dough, such as fructose, also provides an energy advantage since it is reduced to mannitol, favoring the concomitant production of acetate and ATP [46,47].

The physicochemical characteristics of bread samples produced with FSLf, FSLp, and FSc during 6 days of storage were determined. Results showed that produced bread had an acidic environment (pH below 4.5), which is a limiting factor for the growth and survival of spoilage fungi. Free water tends to migrate from the crumb to the crust and from the crust to the environment during bread aging, reducing the bread moisture content, which occurred in low intensity in bread produced with Lf.

A higher specific volume was detected for bread produced with FSLf. The specific volume in bread loaves indicates how capable the dough is of expanding during the rising and baking process, as well as the intensity of the bacterial proteolysis of the gluten network in doughs, resulting in greater extensibility and increased volume in bread loaves. In addition, the moisture loss and shrinkage caused by starch retrogradation could reduce the bread volume during aging [19].

Bread crumb color assessment showed that samples had minor variations in the measured color parameters. Especially, the b* parameter is associated with a slightly yellow crumb, a common characteristic of bread prepared with mixing processes that promote low oxidation of carotenoid pigments in wheat flour [48], which probably was not affected by the type of sourdough used to formulate the bread. Some factors could affect bread texture, including the EPS content, which is associated with the higher specific volume and improvement in the hardness of bread [19]. In this study, the elasticity was less affected during bread aging when FSLp and FSLf were used.

Differential scanning calorimetry (DSC) characterized retrograded starch after baking bread made with freeze-dried sourdough. DSC evaluated whether the use of sourdough previously inoculated with Lp and Lf influenced the cooking and structure of the bread, as well as the thermal stability of the bread loaves [49]. Since enthalpy (H) is the amount of energy spent to carry out a calorimetric phenomenon, it is possible to observe the enthalpy of the fusion of recrystallized amylopectin, thus obtaining the amount of retrograded starch in the bread. The enthalpy variation has been directly proportional to the amount of retrograded starch in the sample [50]. Starch retrogradation is one of the main bread aging agents and the presence of LAB capable of producing EPS and acidifying the sourdough are key factors to delay bread aging [36]. The lowest enthalpy value at day 6 of storage was detected in bread produced with Lf, indicating a low amount of recrystallized starch and maintenance of sensory characteristics.

The in vitro assessment of digestibility showed that bread made with Lp and Lf had the highest digestibility scores. This result suggested that Lp and Lf could improve the proteolytic activity of bread samples during fermentation, including the gluten molecule, resulting in improved digestibility. LAB and yeast fermentation have been previously reported to enhance nutritional content and food digestibility [51,52].

## 5. Conclusions

The results showed the viability of freeze-dried sourdough with *L. pentosus* 129 or *L. fermentum* 139 and their effects on bread fermentation. The use of FOS resulted in a higher survival rate and metabolic activity after the sourdough freeze-drying and high survival of bacteria and yeasts during 60 days of storage. In general, using tested strains improved the microbiological and physicochemical characteristics of sourdough and produced long-term fermentation bread, besides reducing bread aging and increasing digestibility. The freeze-drying protocol, especially combined with *L. fermentum* 139, promoted a satisfactory increase in bread volume without the need for using baker’s yeast, which could represent an innovative contribution to developing powdered sourdough for bread production. These results indicated that freeze-dried sourdoughs containing *L. pentosus* 129 or *L. fermentum* 139 should be feasible approaches to the standardization of the production protocols for the bakery industry, especially for long-fermentation bread production. Further studies should explore the impacts of the developed sourdoughs on the sensory characteristics of bread and the generation of bioactive compounds in this product.

## Figures and Tables

**Table 1:** Acidification capacity (average ± standard deviation, n: 3) for *Lactobacillus* spp. strains in sterile flour broth (SFE) and control (uninoculated) during 72 h (30 ± 1 °C).

Time (Hours)	*Lactobacillus* spp. Strains					
	Control	Lf 56	Lpc 106	Lp 129	Lf 139	Lf 141
Zero	5.65 ± 0.07 ^Ba^	6.41 ± 0.01 ^Aa^	6.67 ± 0.04 ^Aa^	5.58 ± 0.12 ^Ba^	5.78 ± 0.03 ^Ba^	5.80 ± 0.13 ^Ba^
2	5.75 ± 0.07 ^Ba^	6.46 ± 0.08 ^Aa^	6.66 ± 0.09 ^Aa^	5.65 ± 0.07 ^Ba^	5.75 ± 0.07 ^Ba^	5.88 ± 0.11 ^Ba^
4	5.79 ± 0.13 ^Ba^	6.59 ± 0.13 ^Aa^	6.59 ± 0.13 ^Aa^	5.06 ± 0.06 ^Cb^	5.26 ± 0.06 ^BCb^	5.55 ± 0.07 ^Ba^
6	5.78 ± 0.11 ^Ba^	6.48 ± 0.12 ^Aa^	6.55 ± 0.07 ^Aa^	4.58 ± 0.03 ^Cc^	4.67 ± 0.10 ^Cc^	4.78 ± 0.11 ^Cb^
8	5.75 ± 0.07 ^Ba^	6.56 ± 0.08 ^Aa^	5.98 ± 0.03 ^Bb^	4.47 ± 0.04 ^Cc^	4.45 ± 0.08 ^Cc^	4.54 ± 0.08 ^Cb^
24	5.44 ± 0.08 ^Aa^	4.08 ± 0.02 ^Bb^	3.76 ± 0.09 ^Cc^	3.59 ± 0.01 ^Cd^	3.55 ± 0.07 ^Cd^	3.76 ± 0.06 ^Cc^
48	4.35 ± 0.07 ^Ab^	4.03 ± 0.10 ^Bb^	3.47 ± 0.03 ^Cc^	3.57 ± 0.04 ^Cd^	3.59 ± 0.01 ^Cd^	3.69 ± 0.01 ^Cc^
72	3.60 ± 0.01 ^Ac^	3.48 ± 0.02 ^Ac^	3.42 ± 0.11 ^Ad^	3.48 ± 0.03 ^Ad^	3.48 ± 0.03 ^Ad^	3.58 ± 0.03 ^Ac^

^a–d^ Average ± standard with different uppercase lower letters in the same column differ statistically (*p* < 0.05), based on Tukey’s test. ^A–C^ Average ± standard deviation with different uppercase capital letters in the same row differ statistically (*p* < 0.05), based on Tukey’s test. Control: uninoculated SFE; Lf 56: *L. fermentum* 56; Lpc 106: *L. paracasei* 106; Lp 129: *L. pentosus* 129; Lf 139: *L. fermentum* 139; Lf 141: *L. fermentum* 141.

**Table 2:** Viable cell counts of lactic acid bacteria (LAB) and yeasts (log_10_ CFU/g, average ± standard deviation, n: 3) in sourdough during a 10-day propagation period (30 ± 1 °C).

Time (Days)	Sourdough Samples					
	LAB			Yeasts		
	Sc	SLp	SLf	Sc	SLp	SLf
Zero	<2	5.70 ± 0.02 ^Af^	5.44 ± 0.06 ^Bg^	4.93 ± 0.03 ^Ac^	4.49 ± 0.63 ^Abc^	4.81 ± 0.09 ^Ac^
1	4.15 ± 0.21 ^Bf^	6.68 ± 0.28 ^Ae^	6.65 ± 0.24 ^Af^	4.09 ± 0.08 ^Bd^	6.54 ± 0.08 ^Aa^	6.71 ± 0.06 ^Aa^
2	4.00 ± 0.01 ^Bf^	7.22 ± 0.06 ^Ad^	7.23 ± 0.14 ^Ade^	2.98 ± 0.10 ^Bf^	7.08 ± 0.06 ^Aa^	7.08 ± 0.04 ^Aa^
3	8.17 ± 0.01 ^Ad^	5.56 ± 0.36 ^Bf^	5.36 ± 0.26 ^Bg^	2.85 ± 0.08 ^Bf^	4.56 ± 0.10 ^Ac^	3.75 ± 0.08 ^Ad^
4	5.69 ± 0.08 ^Ce^	6.22 ± 0.21 ^Be^	9.16 ± 0.11 ^Aa^	3.28 ± 0.04 ^Be^	2.86 ± 0.06 ^Bc^	3.75 ± 0.04 ^Ad^
5	8.62 ± 0.06 ^Ac^	8.42 ± 0.02 ^Bbc^	7.00 ± 0.02 ^Cef^	2.73 ± 0.10 ^Bf^	3.16 ± 0.43 ^ABc^	3.75 ± 0.10 ^Ad^
6	8.82 ± 0.03 ^Bb^	9.05 ± 0.13 ^Aa^	9.12 ± 0.03 ^Aa^	2.68 ± 0.07 ^Cf^	5.23 ± 0.27 ^Abc^	3.84 ± 0.02 ^Bd^
7	9.19 ± 0.02 ^Aa^	8.24 ± 0.21 ^Bc^	7.59 ± 0.16 ^Ccd^	2.68 ± 0.08 ^Bf^	5.66 ± 0.14 ^Ab^	5.07 ± 0.25 ^Ac^
8	8.50 ± 0.12 ^Ac^	7.97 ± 0.02 ^Bc^	7.77 ± 0.21 ^Bc^	3.98 ± 0.06 ^Bd^	6.83 ± 0.23 ^Aa^	6.05 ± 0.18 ^Ab^
9	9.16 ± 0.15 ^Aa^	8.78 ± 0.05 ^Bab^	8.44 ± 0.06 ^Cb^	6.14 ± 0.37 ^Ab^	7.02 ± 0.22 ^Aa^	6.95 ± 0.29 ^Aa^
10	9.13 ± 0.12 ^Aa^	8.87 ± 0.14 ^Aab^	9.01 ± 0.08 ^Aa^	7.20 ± 0.16 ^Aa^	7.13 ± 0.24 ^Aa^	7.15 ± 0.15 ^Aa^

^a–g^ Average ± standard deviation with different uppercase lower letters in the same column differ statistically (*p* < 0.05), based on Tukey’s test. ^A–C^ Average ± standard deviation with different uppercase capital letters in the same row differ statistically (*p* < 0.05), based on Tukey’s test. Sc: Sourdough control; SLp: Sourdough with *L. pentosus* 129; SLf: Sourdough with *L. fermentum* 139.

**Table 3:** Viable cell counts of lactic acid bacteria (LAB) and yeasts (log CFU/g, average ± standard deviation, n: 3) in freeze-dried sourdough during 60 days of storage (28 ± 1 °C).

	**LAB (CFU/g)**					
**Sourdough Sample**		**Day of Storage**				
	**Before Freeze-Drying**	**0**	**15**	**30**	**45**	**60**
FSc	8.72 ± 0.04 ^Ac^	8.64 ± 0.04 ^Aa^	8.29 ± 0.02 ^Ba^	8.17 ± 0.03 ^Ca^	8.10 ± 0.05 ^Ca^	7.81 ± 0.16 ^Da^
FSLp	8.90 ± 0.08 ^Ab^	8.62 ± 0.20 ^Aa^	7.44 ± 0.06 ^Bc^	7.65 ± 0.07 ^Cc^	6.85 ± 0.18 ^Dc^	6.70 ± 0.02 ^Dc^
FSLf	9.08 ± 0.60 ^Aa^	8.50 ± 0.03 ^Aa^	8.22 ± 0.10 ^Ba^	8.02 ± 0.03 ^Cb^	8.07 ± 0.04 ^Ca^	7.70 ± 0.06 ^Da^
	**Yeasts (CFU/g)**					
FSc	7.29 ± 0.02 ^Abc^	7.16 ± 0.03 ^Aa^	7.12 ± 0.01 ^Ab^	7.08 ± 0.08 ^ABb^	7.04 ± 0.03 ^Bd^	6.99 ± 0.05 ^Ba^
FSLp	7.34 ± 0.04 ^Aab^	7.18 ± 0.12 ^Aa^	6.72 ± 0.04 ^Bc^	5.95 ± 0.02 ^Cc^	5.48 ± 0.04 ^Dc^	5.00 ± 0.03 ^Ed^
FSLf	7.35 ± 0.02 ^Aa^	7.20 ± 0.13 ^Aa^	6.76 ± 0.12 ^Ba^	6.18 ± 0.15 ^Ca^	5.95 ± 0.05 ^Da^	5.30 ± 0.02 ^Ec^

^a–d^ Average ± standard deviation with different superscript small letters in the same column row differ statistically (*p* < 0.05), based on Tukey’s test. ^A–E^ Averages ± standard deviation with different superscripts capital letters in the same row differ statistically (*p* < 0.05), based on Tukey’s test. FSc: freeze-dried sourdough control; FSLp: freeze-dried sourdough inoculated with *L. pentosus* 129; FSLf: freeze-dried sourdough inoculated with *L. fermentum* 139.

**Table 4:** Physicochemical parameters (average ± standard deviation, n: 3) of freeze-dried sourdough inoculated or not with *L. pentosus* 129 (FSLp) and/or *L. fermentum* 139 (FSLf) during 60 days of storage (28 ± 1 °C).

Freeze-Dried Sourdough				
Parameters	Day of Storage	FSc	FSLp	FSLf
pH	0	3.94 ± 0.01 ^Bc^	3.90 ± 0.01 ^Cd^	4.02 ± 0.03 ^Ab^
	15	3.98 ± 0.02 ^Bb^	3.96 ± 0.02 ^Bb^	4.04 ± 0.02 ^Ab^
	30	3.98 ± 0.02 ^Bb^	3.98 ± 0.01 ^Bb^	4.03 ± 0.01 ^Ab^
	45	4.04 ± 0.01 ^Aa^	4.00 ± 0.04 ^Aab^	4.06 ± 0.03 ^Aab^
	60	4.05 ± 0.04 ^Aab^	3.93 ± 0.01 ^Bc^	4.03 ± 0.02 ^Aab^
TTA (mL NaOH/10 g)	0	6.07 ± 0.15 ^Bab^	6.68 ± 0.35 ^Aa^	6.30 ± 0.21 ^ABa^
	15	5.84 ± 0.17 ^ABc^	6.09 ± 0.07 ^Ab^	5.15 ± 0.20 ^Cc^
	30	6.01 ± 0.11 ^Ac^	6.09 ± 0.21 ^Ac^	5.21 ± 0.25 ^Bbc^
	45	6.30 ± 0.15 ^Aa^	5.25 ± 0.14 ^Cd^	5.48 ± 0.06 ^Bb^
	60	5.91 ± 0.25 ^Aabc^	5.71 ± 0.25 ^ABc^	5.15 ± 0.30 ^Cbc^
a_w_	0	0.18 ± 0.00 ^Cc^	0.23 ± 0.02 ^Be^	0.26 ± 0.02 ^Ae^
	15	0.19 ± 0.02 ^Cbc^	0.30 ± 0.01 ^Ad^	0.28 ± 0.01 ^Bd^
	30	0.20 ± 0.01 ^Bb^	0.37 ± 0.00 ^Ac^	0.36 ± 0.01 ^Ac^
	45	0.21 ± 0.01 ^Cb^	0.48 ± 0.02 ^Aa^	0.38 ± 0.00 ^Bb^
	60	0.33 ± 0.00 ^Ca^	0.45 ± 0.01 ^Ab^	0.42 ± 0.01 ^Ba^
Moisture (%)	0	3.81 ± 0.03 ^Bc^	4.53 ± 0.09 ^Ad^	3.66 ± 0.04 ^Cd^
	15	4.19 ± 0.04 ^Bb^	4.62 ± 0.06 ^Cd^	4.95 ± 0.07 ^Ac^
	30	4.41 ± 0.20 ^Cb^	5.21 ± 0.04 ^Ac^	5.08 ± 0.06 ^Bc^
	45	5.07 ± 0.07 ^Ca^	7.12 ± 0.14 ^Ab^	5.91 ± 0.02 ^Bb^
	60	5.16 ± 0.04 ^Ca^	7.96 ± 0.06 ^Aa^	6.67 ± 0.14 ^Ba^

^a–e^ Average ± standard deviation with different letters in the same column differ statistically (*p* < 0.05), based on Tukey’s test. ^A–C^ Average ± standard deviation with different letters in the same row differ statistically (*p* < 0.05), based on Tukey’s test. FSc: freeze-dried sourdough control; FSLp: freeze-dried sourdough inoculated with *L. pentosus* 129; FSLf: freeze-dried sourdough inoculated with *L. fermentum* 139.

**Table 5:** Contents of sugars and organic acids (average ± standard deviation, n: 3) in freeze-dried sourdough inoculated or not with *L. pentosus* 129 (FSLp) or *L. fermentum* 139 (FSLf).

Parameters	Freeze-Dried Sourdough			
		FSc	FSLp	FSLf
Sugars (g/kg)	Fructose	3.58 ± 0.12 ^B^	3.04 ± 0.09 ^C^	5.30 ± 0.17 ^A^
	Glucose	3.90 ± 0.16 ^A^	3.31 ± 0.11 ^B^	1.20 ± 0.09 ^C^
	Maltose	<LOD	<LOD	0.61 ± 0.11 ^A^
Organic Acids (g/kg)	Acetic	<LOD	<LOD	<LOD
	Citric	0.08 ± 0.02 ^A^	0.07 ±0.01 ^A^	0.02 ± 0.04 ^A^
	Formic	0.07 ± 0.03 ^A^	0.04 ± 0.02 ^A^	0.02 ± 0.02 ^A^
	Lactic	0.12 ± 0.19 ^B^	0.22 ± 0.07 ^B^	0.70 ± 0.13 ^A^
	Malic	0.22 ± 0.13 ^A^	0.19 ± 0.10 ^A^	<LOD
	Succinic	1.43 ± 0.25 ^A^	0.30 ± 0.16 ^B^	0.03 ± 0.09 ^C^

^A–C^ Average ± standard deviation with different letters in the same row differ statistically (*p* < 0.05), based on Tukey’s test. FSc: freeze-dried sourdough control; FSLp: freeze-dried sourdough inoculated with *L. pentosus* 129; FSLf: freeze-dried sourdough inoculated with *L. fermentum* 139. <LOD: Below the limit of detection.

**Table 6:** Total titratable acidity (TTA), pH, water activity (a_w_), moisture, specific volume, color, texture [hardness (N), elasticity chewiness, and differential scanning calorimetry (heat of fusion of retrograded starch, ∆H) (average ± standard deviation, n: 3) of long-fermentation bread made with freeze-dried sourdough inoculated or not with *L. pentosus* 129 (FSLp) or *L. fermentum* 139 (FSLf) during 6 days of storage (28 ± 1 °C).

		Bread Sample		
Parameters	Day of Storage	Control	FSLp	FSLf
pH	Zero	4.43 ± 0.06 ^Aa^	4.40 ± 0.00 ^Aa^	4.40 ± 0.00 ^Aa^
	3	4.38 ± 0.02 ^Aa^	4.38 ± 0.02 ^Aa^	4.39 ± 0.03 ^Aa^
	6	4.40 ± 0.00 ^Aa^	4.40 ± 0.00 ^Aa^	4.40 ± 0.00 ^Aa^
TTA (mL NaOH/10 g)	Zero	2.90 ± 0.21 ^Ba^	3.32 ± 0.49 ^ABa^	3.43 ± 0.15 ^Aa^
	3	2.90 ± 0.30 ^Aa^	2.92 ± 0.07 ^Aa^	2.97 ± 0.10 ^Ab^
	6	2.74 ± 0.06 ^Ab^	2.87 ± 0.20 ^Aa^	2.71 ± 0.15 ^Ac^
Moisture (%)	Zero	37.02 ± 0.80 ^Aa^	34.60 ± 0.27 ^Ba^	37.79 ± 0.72 ^Aa^
	3	33.90 ± 0.19 ^Ab^	33.00 ± 0.27 ^Bb^	33.29 ± 0.09 ^Bb^
	6	29.52 ± 0.84 ^Bc^	29.66 ± 0.45 ^Bc^	33.19 ± 0.13 ^Ab^
Specific volume (cm^3^/g)	Zero	2.94 ± 0.08 ^Ba^	2.82 ± 0.08 ^Ba^	3.22 ± 0.16 ^Aa^
	3	2.85 ± 0.08 ^Bab^	2.79 ± 0.16 ^Ba^	3.12 ± 0.04 ^Aab^
	6	2.82 ± 0.02 ^Bb^	2.65 ± 0.14 ^Ca^	2.99 ± 0.08 ^Ab^
Bread crumb color				
L*	Zero	76.63 ± 0.84 ^Aa^	77.94 ± 0.74 ^Aa^	77.93 ± 0.31 ^Ab^
	3	78.56 ± 0.90 ^Aa^	77.41 ± 0.41 ^Ba^	79.59 ± 0.75 ^Aa^
	6	76.88 ± 0.70 ^Ca^	78.12 ± 0.53 ^Ba^	79.82 ± 0.78 ^Aa^
a*	Zero	0.61 ± 0.06 ^ABa^	0.54 ± 0.08 ^Ba^	0.68 ± 0.01 ^Aa^
	3	0.56 ± 0.08 ^Aa^	0.51 ± 0.12 ^Aa^	0.55 ± 0.01 ^Ab^
	6	0.52 ± 0.11 ^Aa^	0.44 ± 0.11 ^Aa^	0.46 ± 0.07 ^Ac^
b*	Zero	14.71 ± 0.41 ^Bc^	14.40 ± 0.57 ^Ba^	16.38 ± 0.87 ^Aa^
	3	16.23 ± 0.64 ^Aa^	14.98 ± 0.28 ^Ba^	15.53 ± 0.33 ^ABa^
	6	15.03 ± 0.17 ^Ab^	15.54 ± 0.91 ^Aa^	14.66 ± 0.29 ^Ab^
Bread texture				
Hardness (N)	Zero	3.68 ± 0.28 ^Ac^	3.61 ± 0.22 ^Ac^	2.36 ±0.16 ^Bc^
	3	8.40 ± 0.27 ^Bb^	9.56 ± 0.25 ^Ab^	5.29 ± 0.16 ^Cb^
	6	13.22 ± 0.56 ^Aa^	13.16 ± 0.29 ^Aa^	10.33 ± 0.47 ^Ba^
Elasticity	Zero	0.45 ± 0.02 ^Aa^	0.43 ± 0.03 ^Aba^	0.43 ± 0.05 ^Aa^
	3	0.29 ± 0.02 ^Ab^	0.27 ± 0.02 ^Ab^	0.27 ± 0.03 ^Ab^
	6	0.23 ± 0.01 ^Ac^	0.27 ± 0.06 ^Ab^	0.22 ± 0.04 ^Ab^
Chewiness (N)	Zero	2.40 ± 0.18 ^Ac^	2.30 ± 0.22 ^Ac^	1.50 ± 0.07 ^Bc^
	3	4.16 ± 0.08 ^Ab^	4.22 ± 0.27 ^Ab^	2.51 ± 0.18 ^Bb^
	6	5.30 ± 0.16 ^Aa^	5.33 ± 0.33 ^Aa^	3.65 ± 0.01 ^Ba^
Differential scanning calorimetry (∆H)	Zero	39.74 ± 0.01 ^Bb^	31.82± 0.01 ^Cb^	43.24 ± 0.02 ^Ab^
	6	86.2 ± 0.02 ^Aa^	73.2 ± 0.01 ^Ba^	61.1± 0.02 ^Ca^

^a–c^ Average ± standard deviation with different superscript small letters in the same column differ statistically (*p* < 0.05), based on Tukey’s test. ^A–C^ Average ± standard deviation with different superscript capital letters differs statistically (*p* < 0.05), based on Tukey’s test.

## Data Availability

All data generated in this article, including Supplementary Materials, are shared in the publication. The corresponding author can provide data sharing upon reasonable request.

## Supplementary Materials

The following supporting information can be downloaded at: https://www.mdpi.com/article/10.3390/microorganisms12061199/s1. Table S1. Description of *Lactobacillus* spp. selected to study the potential use of starter culture in sourdough production. Table S2. Bread formulations with sourdough inoculated with *Lactobacillus* spp. strains and controls. Table S3. Ingredient proportions to produce bread with *Lactobacillus* spp. inoculated sourdough and control samples. Table S4. Production of exopolysaccharide (EPS) (average ± standard deviation n: 3) by *Lactobacillus* spp. strains. Table S5. Differential scanning calorimetry (DSC), initial transition temperature (T0), peak transition temperature (Tp), and final transition temperature (TF) of long-fermentation bread made with freeze-dried sourdough inoculated or not with *L. pentosus* 129 and/or *L. fermentum* 139 at zero and 6 days of storage (28 ± 1°C). Figure S1. Principal Component Analysis (PCA) for selecting Lactobacillus spp. strains based on the acidification and EPS production capacity.
