# Supplementary material for: Exploring the Effects of Freeze-Dried Sourdoughs with Lactiplantibacillus pentosus 129 and Limosilactobacillus fermentum 139 on the Quality of Long-Fermentation Bread
Source: Microorganisms. 2024 Jun 14;12(6):1199. doi: 10.3390/microorganisms12061199 (PMC11205311; doi:10.3390/microorganisms12061199)
Supplement: Supplementary file 1 [file microorganisms-12-01199-s001.zip › microorganisms-3009712-supplementary.pdf]

Table S1. Description of *Lactobacillus* spp. selected to study the potential use of starter culture in sourdough production.

Strain	Code	Origin	Reference
Limosilactobacillus fermentum 56	Lf 56	Acerola by-product (*Malpighia glabra* L.)	
Lacticaseibacillus paracasei 106	Lpc 106	Graviola by-product (*Annona muricata* L.)	
Lactiplantibacillus pentosus 129	Lp 129	Mango by-product (*Mangifera indica* L.)	[29,30]
Limosilactobacillus fermentum 139	Lf 139	Mango by-product (*Mangifera indica* L.)	
Limosilactobacillus fermentum 141	Lf 141	Mango by-product (*Mangifera indica* L.)	

Table S2. Bread formulations with sourdough inoculated with *Lactobacillus* spp. strains and controls.

Code	*Lactobacillus* spp.	Freeze-dried
Sc	No	No
SLp	Yes – Strain Lp 129	No
SLf	Yes – Strain Lf 139	No
FSc	No	Yes
FSLp	Yes – Strain Lp 129	Yes
FSLf	Yes – Strain Lf 139	Yes

Table S3. Ingredient proportions to produce bread with *Lactobacillus* spp. inoculated sourdough and control samples.

	Formulation (%)
White wheat flour	100
Water	55
Sugar	4
Salt	1.5
Butter	2
Sourdough	40

Table S4. Production of exopolysaccharide (EPS) (average ± standard deviation n: 3) by *Lactobacillus* spp. strains.

**LAB Strains**	**EPS (mg/L)**
Lf 56	0.33±0.03^{a}
Lpc 106	0.16±0.03^{b}
Lp 129	0.20±0.01^{b}
Lf 139	0.20±0.06^{b}
Lf 141	0.13±0.01^{b}

$^{a-b}$ Average ± standard deviation with different superscript letters differs ($p < 0.05$), based on Tukey's test.

* Proteolytic activity considered (+) when halo detected ≥ 1mm. Lf 56: *L. fermentum* 56; Lpc 106: *L. paracasei* 106; Lp 129: *L. pentosus* 129; Lf 139: *L. fermentum* 139; Lf 141: *L. fermentum* 141.

Table S5. Differential scanning calorimetry (DSC), initial transition temperature (T0), peak transition temperature (Tp), and final transition temperature (TF) of long-fermentation bread made with freeze-dried sourdough inoculated or not with *L. pentosus* 129 and/or *L. fermentum* 139 at zero and 6 days of storage (28 ± 1°C).

Bread	Day of storage	T_0 (°C)	T_F (°C)	T_P (°C)
BFSc	Zero	26.9	50.6	35.0
BFSLp		26.4	49.8	34.0
BFSLf		28.3	55.5	37.1
BFSc	6	23.1	32.3	49.9
BFSLp		24.3	39.6	47.0
BFSLf		24.2	32.4	43.2

BFSc: Bread Control, sourdough bread (wild microbiota); BFSLp: bread with with freeze-dried sourdough inoculated with *L. pentosus* 129; BFSLf: bread with freeze-dried sourdough inoculated with *L. fermentum 139*.

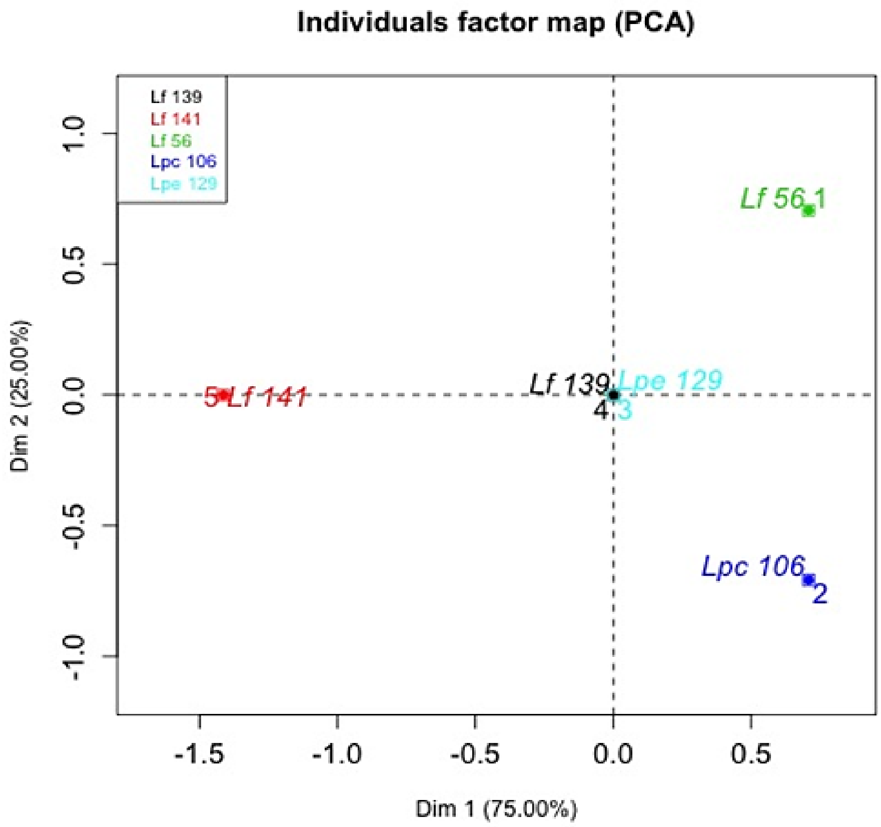

Figure S1. Principal Component Analysis (PCA) for selecting Lactobacillus spp. strains based on the acidification and EPS production capacity.
